# Photo-isolation chemistry for high-resolution and deep spatial transcriptome with mouse tissue sections

**DOI:** 10.1016/j.xpro.2022.101346

**Published:** 2022-04-22

**Authors:** Mizuki Honda, Ryuichi Kimura, Akihito Harada, Kazumitsu Maehara, Kaori Tanaka, Yasuyuki Ohkawa, Shinya Oki

**Affiliations:** 1 Department of Drug Discovery Medicine, Kyoto University Graduate School of Medicine, Kyoto 606-8607, Japan; 2 Division of Transcriptomics, Medical Institute of Bioregulation, Kyushu University, Fukuoka 812-0054, Japan; 3 Precursory Research for Embryonic Science and Technology (PRESTO), Japan Science and Technology Agency (JST), Kawaguchi, Saitama 332-0012, Japan

**Keywords:** Gene Expression, Molecular Biology, Molecular/Chemical Probes, RNAseq, Sequence analysis, Sequencing

## Abstract

Photo-isolation chemistry (PIC) enables isolation of transcriptome information from locally defined areas by photo-irradiation. Here, we present an optimized PIC protocol for formalin-fixed frozen and paraffin mouse sections and fresh-frozen mouse sections. We describe tissue section preparation and permeabilization, followed by *in situ* reverse transcription using photo-caged primers. We then detail immunostaining and UV-mediated uncaging to the target areas, followed by linear amplification of uncaged cDNAs, library preparation, and quantification. This protocol can be applied to various animal tissue types.

For complete details on the use and execution of this protocol, please refer to Honda et al. (2021).

## Highlights

• Preparation of tissue sections and permeabilization
• *In situ* reverse transcription using photo-caged primers
• Immunostaining and UV irradiation to the ROIs
• Linear amplification of uncaged cDNAs before sequencing

## Graphical abstract

**Figure:**
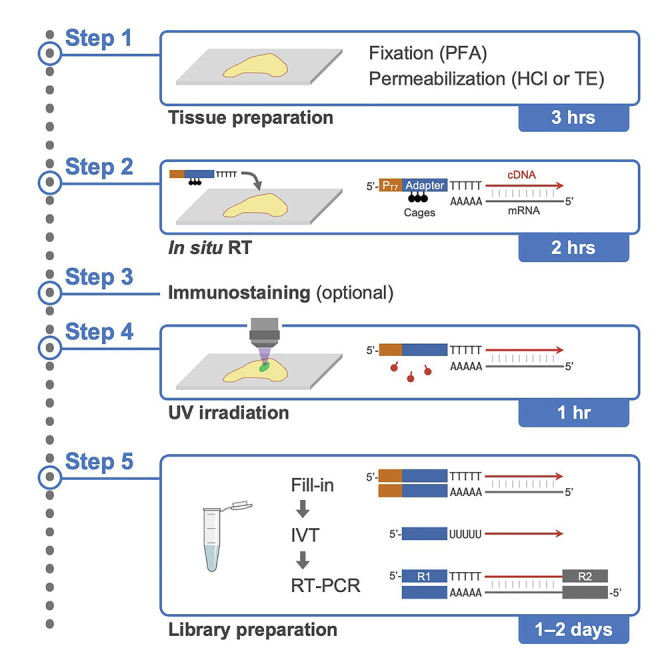

## Before you begin

Photo-isolation chemistry (PIC) is a spatial transcriptome method for locally defined areas with the advantages of high spatial resolution and detection depth, ranging from large to small areas such as brain fields, embryonic tissues, single cells, and intracellular structures. PIC comprises the following five processes: (1) Sections are prepared from fresh or fixed tissues and permeabilized with HCl or Tris-EDTA (TE); (2) mRNAs are reverse-transcribed *in situ* with photo-caged primers; (3) the regions of interest (ROIs) are labeled by immunostaining and/or nuclear staining; (4) cDNA-conjugated primers are uncaged by photo-irradiation; (5) uncaged cDNAs are selectively amplified by *in vitro* transcription (IVT) before sequencing. Once tissue sections are prepared, two days are required to prepare sequencing libraries with standard molecular biology equipment and expertise.

### Tissue preparation

**Figure 1:**
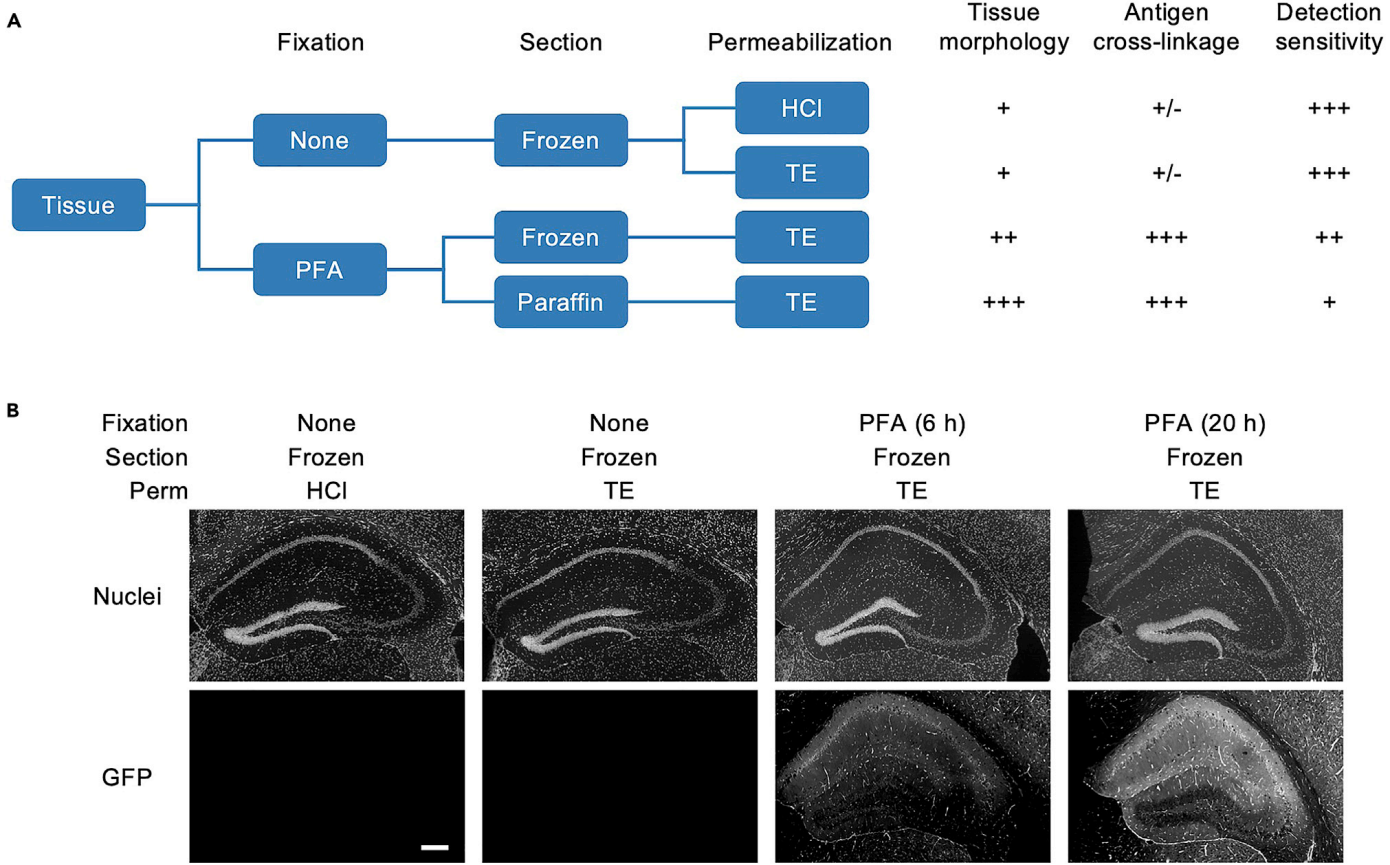
Selection guide for fixation and permeabilization (A) The selection chart for tissue fixation and permeabilization. The fresh-frozen section provides the greatest efficiency for *in situ* RT when permeabilized with HCl or TE, but is inferior to the fixed samples in terms of preservation of tissue morphology and antigen cross-linkage. Formalin-fixed frozen or paraffin sections preserve tissue morphology and antigen cross-linkage for immunolabeling to a greater extent. (B) Brain sections from the hippocampal region in GFP-expressing mice are shown to explain the importance of antigen cross-linkage. GFP was mostly lost in fresh-frozen sections after HCl or TE permeabilization, but preserved in tissues with longer formalin fixation, even after heating with TE. Scale bars, 200 μm.

The efficiency of *in situ* reverse transcription (RT) is best with fresh-frozen sections, followed by formalin-fixed frozen and paraffin sections (Figure 1A). However, formalin-fixed tissues have greater advantages in preserving tissue morphologies and preventing the dissipative loss of marker molecules for the visualization of ROIs, such as immunostaining and reporter protein antigens. In our experience, proteins that are less interactive with other molecules, such as GFP, are lost easily and need to be fixed (Figure 1B), whereas transcription factors and the proteins composing liquid-liquid phase separation are often detected without fixation. Furthermore, because RNAs are bound by various proteins and covalently cross-linked by formalin fixation, a permeabilization step is necessary to eliminate these interferences before *in situ* RT. In our experience, briefly soaking the sections in HCl solution or heating them in TE solution is effective for fresh-frozen sections, whereas only the latter is effective for formalin-fixed tissues. Therefore, with a tissue for the first trial, both unfixed and fixed frozen sections were prepared and permeabilized with HCl or TE before immunostaining for ROI labeling (Figure 1A). If ROIs can be identified simply by nuclear staining, the fresh-frozen section is the best choice. If only paraffin sections were available, permeabilization with TE alone was tested. Therefore, the optimal conditions should be selected by evaluating the balance of tissue morphology, preservation of marker antigens, and efficiency of *in situ* RT.

### Synthesis of caged RT primer

**Figure 2:**
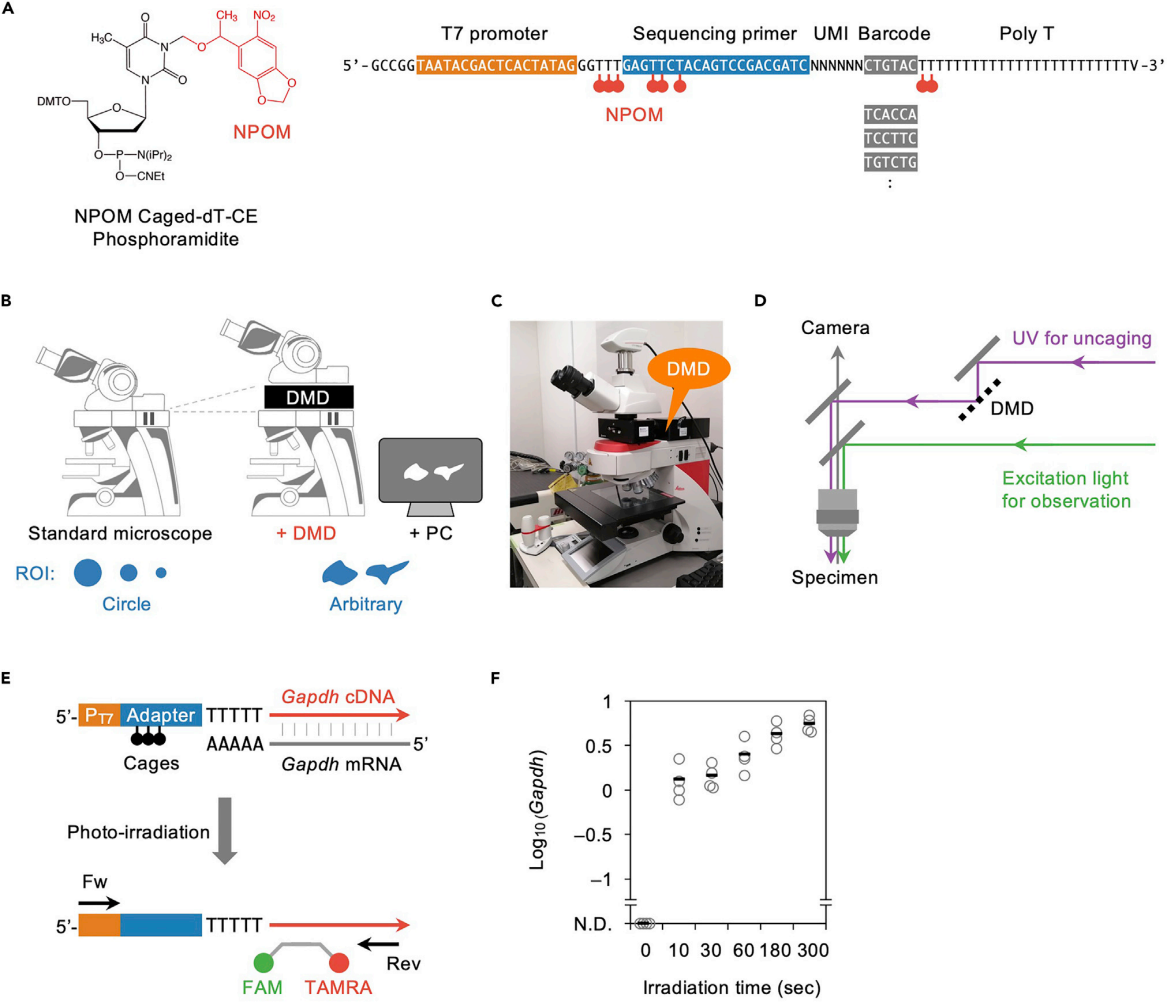
Setting up the photo-irradiation system (A) The structure of NPOM-caged dT and the sequence of NPOM-caged RT primers with multiple barcodes. (B–D) Only circle-shaped light is emitted by a standard fluorescent microscope, but arbitrary shaped light is available when using a DMD (B), which can be extrapolated to most microscopes (C) to irradiate the UV for uncaging through a light path distinct from that originally supplied with the microscope (D). (E and F) Experiments to optimize the irradiation time. The degree of uncaging is simply quantified using TaqMan qPCR and *Gapdh* (E), where given tissues are subjected to *in situ* RT with caged RT primers, photo-irradiated during various periods, and *Gapdh* is quantified using TaqMan qPCR to determine the necessary and sufficient irradiation time for uncaging.

Purchase 50 μmol of 6-nitropiperonyloxymethyl NPOM-caged dT-CE phosphoramidite (Glen Research; #10-1534-95; Young et al., 2009) per construct and send it to the oligo house to synthesize caged RT primers, with the sequence shown in Figure 2A, by which at least 2 O.D. (≃66 μg) of the caged RT primers will be obtained. When caged RT primers with various barcode sequences, multiple samples can be pooled after photo-irradiation and cell lysis, thus reducing the cost of library synthesis for multiple samples.

**Figure 3:**
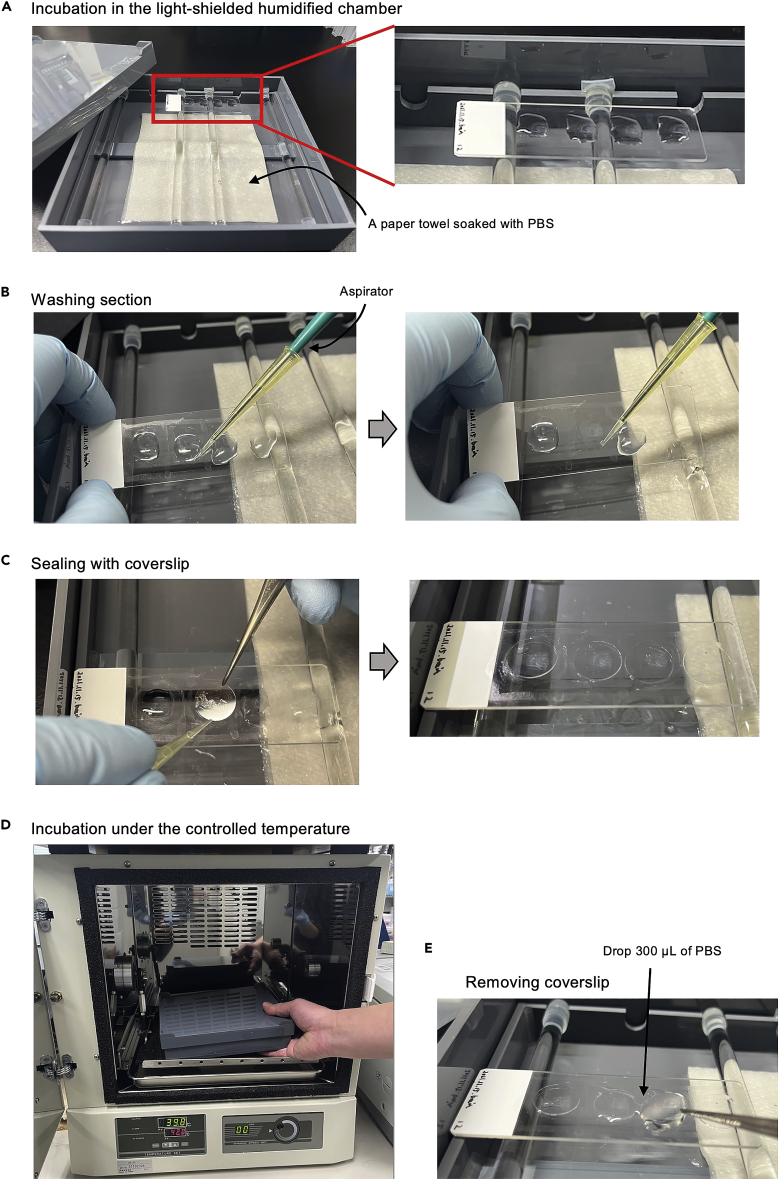
Images of critical handling (A) A Light-shielded and PBS-humidified chamber is used for long time incubation of the tissues such as for *in situ* RT and immunostaining. (B) Tissue sections are washed by dropping and aspirating PBS. (C) Sections are sealed with coverslip using tweezers and pipette tip, taking care that no air bubbles are present on the section. (D) A hybridization oven is used for temperature-controlled incubation for *in situ* RT and tissue lysis reaction. (E) It is easy to remove the coverslips by dropping 300 μL of PBS onto the glass slides, so that the coverslip floats and can be pinched with tweezers.

**CRITICAL:** HPLC purification must not be ordered because UV is used for size evaluation and uncages NPOM moieties; rather, an oligonucleotide purification cartridge (OPC) grade should be ordered for purification. It is preferable to order it as a dried product in a light-shielded tube. After receiving, the product can be stored at −20°C in light-shielded storage even when dissolved in 500 ng/μL with nuclease-free water. In later experiments, samples should be shielded from light during extended incubation times (Figure 3A), such as *in situ* RT and antibody reactions, but other than that, it is acceptable to perform the experiment under normal fluorescent light.

### Examination of irradiation time and cell number

Because NPOM moieties are dissociated at 365 nm as their maximum absorption wavelength, they can be uncaged by the light path and filter cubes often used to excite DAPI and Hoechst under a standard fluorescence microscope, but the light emitted from the objective lens is limited to a circular shape (Figure 2B). To narrow down the irradiation area, it is better to use a higher magnification objective lens. For example, the Leica DM6B can emit a φ250 μm excitation light with a 100× objective lens and can be narrowed down to φ16 μm using fluorescent field diaphragms, which are usually equipped with a standard fluorescence microscope. In contrast, a Digital mirror device (DMD) is appropriate for emitting light of an arbitrary shape according to the drawings on a computer (Figure 2B). We used Mightex Polygon1000-G, which has an excellent resolution by integrating a million micro-mirrors. Polygon1000-G can be extrapolated to upright or inverted microscopes from Leica, Zeiss, Olympus, and Nikon. We used a Leica DM6B upright microscope by mounting the Polygon1000-G between the lens barrel and the epi-illuminator module (Figure 2C). The light source of the DMD was separated from that originally supplied in the microscope to excite the probes for immunofluorescence (Figure 2D). The light source appropriate for the DMD-assisted uncaging is a high-power LED emitting around 365 nm, for which we used a product from Prizmatix (UHP-F-365 LED; 3 W). The above combination of microscope, DMD, and light source we have adopted is sufficient for uncaging with up to 3 min of irradiation. With other combinations, optimization of uncaging conditions is required, which can be easily tested using qPCR without sequencing. For instance, in our first setup, brain sections from adult mice were subjected to *in situ* RT with caged RT primers, and DMD-assisted UV irradiation was performed for 0, 10, 30, 60, 180, or 300 s with a 10× objective at maximum light power. Subsequently, tissue lysates were analyzed using TaqMan qPCR with primers against the T7 promoter and Gapdh 3′ UTR (Figure 2E), and sufficiently detected with photo-irradiation for longer than 3 min (Figure 2F).

### Library synthesis, sequencing, and data analysis

After photo-irradiation, library synthesis in PIC method is largely based on the workflow CEL-seq2 (Hashimshony et al., 2016), one of the most sensitive single-cell RNA-seq technologies. The total tissue lysate was collected and purified. Second strand DNA was synthesized using the nick translation method using RNase H, DNA polymerase I, and a DNA ligase mix, by which only the cDNAs derived from the photo-irradiated region are filled-in to the 5′ end of the T7 promoter, thereby allowing the cDNAs to be linearly amplified as RNAs (aRNAs) via IVT reaction. The libraries were further reverse-transcribed, followed by paired-end sequencing on the Illumina platform (Read 1: UMI and barcode, Read 2: cDNA). The given sequences were separated by the sample barcodes with UMI-tools and mapped to the reference genome using HISAT2. featureCounts and UMI-tools were used to generate UMI count data assigned to genes, and DESeq2 was used to extract DEGs.

### Institutional permissions

All research and animal care procedures were approved by Kyoto University Graduate School of Medicine Institutional Animal Care.

## Key resources table

**Table:** 

REAGENT or RESOURCE	SOURCE	IDENTIFIER
**Chemicals, peptides, and recombinant proteins**		

Nuclease-free water	Nacalai Tesque	Cat#06442-95
Distilled Water	Nacalai Tesque	Cat#49506-64
D-Limonene	Nacalai Tesque	Cat#09480-63
Phosphate buffered saline (PBS) tablets (pH 7.4)	Takara Bio	Cat#T9181
16% formaldehyde (w/v), methanol-free	Electron Microscopy Sciences	Cat#15710
Tris-EDTA buffer solution (TE buffer; pH 8.0)	Nacalai Tesque	Cat#32739-31
Triton X-100	Nacalai Tesque	Cat#35501-02
5 N HCl	Nacalai Tesque	Cat#7647-01-0
1 M Tris-HCl (pH 8.0)	Nacalai Tesque	Cat#06938-15
0.1% tTBS (10× TBST; pH 7.4)	Nacalai Tesque	Cat#12750-81
0.1 M DTT	Thermo Fisher Scientific	Cat#Y00147
Tween-20	Nacalai Tesque	Cat#28353-85
Proteinase K solution (20 mg/mL)	Kanto Chemical Co., Inc.	Cat#34060-96
5 M NaCl	Nacalai Tesque	Cat#06900-14
Polyethylene glycol 8,000 (50% w/v)	Nacalai Tesque	Cat#26065-54
Potassium Acetate	Nacalai Tesque	Cat#28404-15
Magnesium acetate tetrahydrate	Nacalai Tesque	Cat#20821-85
0.5 M EDTA (pH 8.0)	Nacalai Tesque	Cat#06894-85
Ethanol	Nacalai Tesque	Cat#14713-95
NPOM-caged-dT-CE Phosphoramidite	Glen Research	Cat#10-1534-95

**Critical commercial assay**		

SuperScript II reverse transcriptase (200 U/μL)	Thermo Fisher Scientific	Cat#18064071
5× First Strand Buffer	Thermo Fisher Scientific	Cat#Y00146
RNaseOUT (40 U/μL)	Thermo Fisher Scientific	Cat#10777019
dNTP (10 mM each)	New England Biolabs	Cat#N0447L
Qiagen MinElute PCR Purification kit	QIAGEN	Cat#28006
5× Second-Strand Buffer	Thermo Fisher Scientific	Cat#10812014
*E. coli* DNA polymerase I (10 U/μL)	Thermo Fisher Scientific	Cat#18010025
*E. coli* DNA ligase (10 U/μL)	Thermo Fisher Scientific	Cat#18052019
RNase H (2 U/μL)	Thermo Fisher Scientific	Cat#18021071
MEGAscript T7 Transcription Kit	Thermo Fisher Scientific	Cat#AMB13345
ATP (from the MEGAscript T7 Transcription Kit)	Thermo Fisher Scientific	Cat#B8114G5
GTP (from the MEGAscript T7 Transcription Kit)	Thermo Fisher Scientific	Cat#B8134G5
CTP (from the MEGAscript T7 Transcription Kit)	Thermo Fisher Scientific	Cat#B8124G5
UTP (from the MEGAscript T7 Transcription Kit)	Thermo Fisher Scientific	Cat#B8144G5
10× T7 reaction buffer (from the MEGAscript T7 Transcription Kit)	Thermo Fisher Scientific	Cat#B8156G5
10× Enzyme Mix (from the MEGAscript T7 Transcription Kit)	Thermo Fisher Scientific	Cat#B2720G5
TURBO DNase (from the MEGAscript T7 Transcription Kit)	Thermo Fisher Scientific	Cat#B2238G5
ExoSAP-IT Express PCR Product Cleanup	Thermo Fisher Scientific	Cat#75001.1.ML
AMPure XP beads	Beckman Coulter	Cat#A63881
RNAClean XP beads	Beckman Coulter	Cat#A63987
Phusion High-Fidelity PCR Master Mix	New England Biolabs	Cat#M0531L
High Sensitivity DNA Kit	Agilent Technologies	Cat#506704626
Ribonuclease A solution (10 mg/mL)	Nacalai Tesque	Cat#30100-31
Blocking One-P	Nacalai Tesque	Cat#05999-84
Nuclear Violet LCS1	AAT Bioquest	Cat#17543
PhiX Control v3	Illumina	Cat#FC-110-3001
SlowFade Diamond	Thermo Fisher Scientific	Cat#S36963

**Experimental models: Organisms/strains**		

Mouse: Slc:ICR, male, 8 weeks old	Charles River Laboratories	N/A

**Biological samples**		

Adult mouse brains (Slc:ICR, male, 8 weeks old)	Charles River Laboratories	N/A

**Oligonucleotides**		

NPOM-caged RT primer: GCCGGTAA TACGACTCACTATAGGGtttGAGttCt ACAGTCCGACGATCNNNNNNCTGT ACttTTTTTTTTTTTTTTTTTTTTTTV	Honda et al. (2021)	t = NPOM-caged dT
Read2+N6 primer: GCCTTGGCACCCG AGAATTCCANNNNNN	Honda et al. (2021)	N/A
Read1 Index primer: AATGATACGGCGA CCACCGAGATCTACAC[ACTGAGTT] GTTCAGAGTTCTACAGTCCGA	Honda et al. (2021)	Replace [ACTGAGTT] with an appropriate index
Read2 Index primer: CAAGCAGAAGACG GCATACGAGAT[AAGCCTTC] GTGACTGGAGTTCCTTGGCACCCGAG AATTCCA	Honda et al. (2021)	Replace [AAGCCTTC] with an appropriate index
T7 primer for TaqMan qPCR: GCCGGTA ATACGACTCACTATAGG	Honda et al. (2021)	N/A
Reverse primer for mGapdh: CAGCAAG GACACTGAGCAAG	Honda et al. (2021)	N/A
TaqMan probe for mGapdh: FAM- GTGGGTGCAGCGAACTTTATTGA-TAMRA	Honda et al. (2021)	N/A

**Software and algorithms**		

UMI-tools (version 1.0.0)	Smith et al. (2017)	https://github.com/CGATOxford/UMI-tools
Trim Galore! (version 0.6.6)	The Babraham Bioinformatics group	https://www.bioinformatics.babraham.ac.uk/projects/trim_galore/
HISAT2 (version 2.1.0)	Kim et al. (2019)	http://daehwankimlab.github.io/hisat2/
featureCounts (version 1.6.4)	Liao et al. (2014)	http://subread.sourceforge.net
R (version 3.5.2)	CRAN R project	https://www.r-project.org
DESeq2 (version 1.20.0)	Love et al. (2014)	https://bioconductor.org/packages/release/bioc/html/DESeq2.html

**Deposited data**		

Dataset	This paper	Gene Expression Omnibus (GEO) under accession code GSE195459

**Other**		

MAS-coated glass slides	MATSUNAMI	Cat#MAS-01
Round coverslip (φ15 mm)	MATSUNAMI	Cat#C015001
PAP PEN	BMS	Cat#BC-PAPPEN-S
Humidified chamber	Cosmo Bio Co., Ltd	Cat#10HTLS
Aspirator	AXEL	Cat#1-6198-01
Hybridization oven	TAITEC	Cat#HB-80
Fluorescence microscope	Leica Microsystems	Cat#DM6B
Digital mirror device (DMD)	Mightex Systems	Cat#Polygon1000-G
Dichroic mirror for DMD	Semrock	Cat#Di02-R442-25×36
LED light source for DMD	Prizmatix	Cat#UHP-F-365LED
Bioanalyzer	Agilent Technologies	Cat#2100
NovaSeq 6000 System	Illumina	Cat#200012850

## Materials and equipment

**Table:** Blocking solution

Reagent	Final concentration	Amount
Blocking One-P	0.5×	0.5 mL
1× TBST	0.5×	0.5 mL
**Total**	**n/a**	**1 mL**

Freshly prepare before use.

**Table:** Beads binding buffer

Reagent	Final concentration	Amount
50% w/v polyethylene glycol 8,000	20%	4 mL
5 M NaCl	2.5 M	5 mL
Nuclease-free water	n/a	1 mL
**Total**	**n/a**	**10 mL**

This can be stored at room temperature (23°C–25°C) for at least 1 year.

**Table:** Fragmentation Buffer

Reagent	Final concentration	Amount
1 M KOAc	0.5 M	5 mL
1.5 M MgOAc	0.15 M	1 mL
1 M Tris-HCl (pH 8.0)	0.2 M	2 mL
Nuclease-free water	n/a	2 mL
**Total**	**n/a**	**10 mL**

This can be stored at room temperature (23°C–25°C) for at least 1 year.

**Table:** Read1 and 2 Index primer (10 μM each)

Reagent	Final concentration	Amount
100 μM Read1 Index primer	10 μM	10 μL
100 μM Read2 Index primer	10 μM	10 μL
Nuclease-free water	n/a	80 μL
**Total**	**n/a**	**100 μL**

This can be stored at −20°C for at least 1 year.

**Table:** NPOM-caged RT primer mix

Reagent	Final concentration	Amount per section
Nuclease-free water	n/a	5 μL
NPOM-caged RT primer (500 ng/μL)	25 ng/μL	0.5 μL
dNTP mix (10 mM each)	0.5 mM	0.5 μL
**Total**	**n/a**	**6 μL**

Freshly prepare before use.

**Table:** First strand mix

Reagent	Final concentration	Amount per section
5× First Strand Buffer	1×	2 μL
0.1 M DTT	0.01 M	1 μL
RNaseOUT (40 U/μL)	2 U/μL	0.5 μL
SuperScript II Reverse Transcriptase (200 U/μL)	10 U/μL	0.5 μL
**Total**	**n/a**	**4 μL**

Freshly prepare before use.

**Table:** Second-strand mix

Reagent	Final concentration	Amount per tube
5× First Strand Buffer	0.5×	2 μL
5× Second-Strand Buffer	0.5775×	2.31 μL
dNTP mix (10 mM each)	0.115 mM	0.23 μL
*E. coli* DNA Polymerase I (10 U/μL)	0.15 U/μL	0.3 μL
*E. coli* DNA Ligase (10 U/μL)	0.04 U/μL	0.08 μL
RNase H (2 U/μL)	0.008 U/μL	0.08 μL
**Total**	**n/a**	**5 μL**

Freshly prepare before use.

**Table:** IVT mix

Reagent	Final concentration	Amount per tube
ATP	7.5 mM	1.6 μL
GTP	7.5 mM	1.6 μL
CTP	7.5 mM	1.6 μL
UTP	7.5 mM	1.6 μL
10× T7 reaction buffer	1×	1.6 μL
10× Enzyme Mix	1×	1.6 μL
**Total**	**n/a**	**9.6 μL**

Freshly prepare before use.

**Table:** aRNA–N6 primer mix

Reagent	Final concentration	Amount per tube
aRNA (step 83)	n/a	4.5 μL
Read2+N6 primer (250 ng/μL)	25 ng/μL	1 μL
dNTP mix (10 mM each)	0.5 mM each	0.5 μL
**Total**	**n/a**	**6 μL**

Freshly prepare before use.

**Table:** Reverse transcription mix

Reagent	Final concentration	Amount per tube
5× First Strand Buffer	1×	2 μL
0.1 M DTT	0.01 M	1 μL
RNaseOUT (40 U/μL)	2 U/μL	0.5 μL
SuperScript II Reverse Transcriptase (200 U/μL)	10 U/μL	0.5 μL
**Total**	**n/a**	**4 μL**

Freshly prepare before use.

**Table:** Library PCR mix

Reagent	Final concentration	Amount per tube
Nuclease-free water	n/a	10.4 μL
Read1 and 2 - Index primer (10 μM each)	0.8 μM each	3.6 μL
Phusion High-Fidelity PCR Master Mix (2×)	1×	22.5 μL
Secondary RT products (step 89)	n/a	8.5 μL
**Total**	**n/a**	**45 μL**

Freshly prepare before use.

## Step-by-step method details

### Post-fixation of tissue sections

**Timing: 15–80 min**

Before performing PIC experiments, prepare fresh-frozen sections or formalin-fixed frozen or paraffin sections at a thickness of 10 μm on MAS-coated glass slides. When using PFA and D-limonene, work in a fume hood.

1. The tissues are hydrated, washed, and fixed using PFA solution according to the following protocols for frozen (a) or paraffin (b) sections:
  **CRITICAL:** The liquid volume of PBS or PFA dropped on the sections depends on the size of the hydrophobic barrier surrounded by PAP PEN. For a 1 × 1 cm hydrophobic barrier, a volume of 50 μL of fluid is optimal to fully cover the sections and prevent leakage from the barrier. The following protocols describe a 1 × 1 cm hydrophobic barrier. Use an aspirator with a fine needle to remove the liquid.
  a. Frozen sections (≈15 min).
    i. Drop and aspirate 50 μL of PBS onto the section twice to remove the OCT compound (Figure 3B).
    ii. Drop 50 μL of 4% PFA in PBS onto the section and incubate at RT for 10 min.
    iii. Aspirate the PFA solution and wash tissue sections twice with 50 μL of PBS.
  b. Paraffin sections (≈80 min).
    i. Immerse the sections in a glass staining jar containing D-Limonene and incubate at RT for 5 min.
    ii. Repeat step (i) two more times with a separate glass staining jar.
    iii. Immerse sections in a glass staining jar containing ethanol (96%–100% [v/v]) and incubate at RT for 5 min.
    iv. Repeat step (iii) two more times with a separate glass staining jar.
    v. Immerse the sections in a glass staining jar containing 90% ethanol and incubate at RT for 5 min.
    vi. Immerse the sections in a glass staining jar containing 80% ethanol and incubate at RT for 5 min.
    vii. Immerse the sections in a glass staining jar containing nuclease-free water and incubate at RT for 15 min.
    viii. Remove glass slides from water, aspirate remaining water, and create a hydrophobic barrier around the section using PAP PEN.
    ix. Drop 50 μL of PBS onto the sections.
    x. Aspirate the PBS, drop 50 μL of 4% PFA in PBS onto the section and incubate at RT for 10 min.
    xi. Aspirate the PFA solution and wash the tissue sections twice with 50 μL of PBS.

### Permeabilization

**Timing: 35–75 min**

Permeabilization step is critical to enhancing the efficiency of *in situ* RT, as mentioned in “tissue preparation” of before you begin and Figure 1. Subsequent steps are performed on a laboratory bench suitable for molecular biological experiments.

2. Permeabilize sections with HCl (a) or TE (b) are as follows:
  a. HCl treatment (≈35 min).
    i. Aspirate PBS from the sections, drop 25 μL of 5% (v/v) Triton X-100 in PBS onto the section, and incubate at RT for 3 min.
      **CRITICAL:** The drop of 25 μL of Triton X-100 is the optimal volume to prevent leakage from a 1 × 1 cm hydrophobic barrier.
    ii. Aspirate Triton X-100 in PBS, wash the tissue sections twice with 50 μL of PBS.
    iii. Aspirate PBS, drop 50 μL of 0.1 N HCl in nuclease-free water on the section, and incubate at RT for 5 min.
    iv. Aspirate the HCl solution, drop 50 μL of 1 M Tris-HCl (pH 8.0) onto the section, and incubate at RT for 5 min.
    v. Aspirate the Tris solution, immerse the sections in preheated PBS at 65°C in a polypropylene staining jar and incubate in a hybridization oven for 5 min to extend the RNAs.
    vi. Immerse the sections in a polypropylene staining jar containing ice-cold PBS for at least 3 min until the sections are fully cooled.
    vii. Place a paper towel on the bottom of the humidified chamber and add 40 mL of PBS to balance the vapor pressure (Figure 3A).
      **CRITICAL:** Do not humidify with nuclease-free water. The vapor of the water will be absorbed by the higher salt solution loaded onto the sections, causing leakage from the hydrophobic barriers during incubation.
    viii. Remove the glass slides from the PBS, aspirate remaining PBS, and place the glass slides in the humidified chamber.
    ix. Wash the tissue sections twice with 50 μL of PBS.
  b. TE treatment (≈75 min).
    i. Aspirate the PBS from sections, immerse the sections in TE buffer (pH 8.0) preheated to 70°C in a polypropylene staining jar and incubate in a hybridization oven for 1 h.
    ii. Immerse the sections in a polypropylene staining jar containing chilled PBS for at least 3 min until the sections are fully cooled.
    iii. Place a paper towel on the bottom of the humidified chamber and add 40 mL of PBS to balance the vapor pressure.
    iv. Remove the glass slides from the PBS, aspirate remaining PBS, and place the glass slides in the humidified chamber.
    v. Wash the tissue sections twice with 50 μL of PBS (Figure 3B).

### *In situ* RT

**Timing: approximately 1 h**

mRNAs are reverse-transcribed *in situ* with NPOM-caged RT primers.

3. Prepare the NPOM-caged RT primer mix, shown in materials and equipment.

**CRITICAL:** NPOM is stable under indoor fluorescent light, but take care not to leave caged primers under the light for extended periods of time, unless required. By preparing RT primers with multiple barcodes, different samples can be pooled after cell lysis (step 26).

4. After spinning down the sample tube (1,400 × *g*, RT, 2 s), incubate the tubes at 65°C in a preheated thermal cycler for 5 min to relax the secondary structure of the primers and immediately place the tubes on ice for at least 2 min.
5. Prepare the first strand mix, as shown in materials and equipment.
6. Mix 6 μL of NPOM-caged RT primer mix (step 4) and 4 μL of first strand mix (step 5) in a 0.2 mL 8-strip PCR tube by gentle pipetting.
7. Add 10 μL of the NPOM-caged RT primer and the first strand mix (step 6) onto the section and seal with a round coverslip (φ15 mm) (Figure 3C).

**CRITICAL:** Ensure there are no air bubbles present on the section. The size of the coverslip can be changed according to the size of the section, with an equivalent size being the best option.

8. Place the sections in a PBS humidified chamber, and incubate at 42°C for 1 h in a hybridization oven (Figure 3D).
9. Drop 300 μL of PBS onto the coverslip and remove the coverslips using tweezers (Figure 3E).

**CRITICAL:** Be careful not to damage or peel tissue samples. The coverslips will float by dropping PBS onto the glass slides.

10. Immerse sections in PBS preheated to 70°C in a polypropylene staining jar and incubate in a hybridization oven for 10 min to terminate RT reaction.
11. Immerse the sections in a polypropylene staining jar containing chilled PBS for at least 3 min until the sections are fully cooled.

**Pause point:** Sections can be stored at 4°C for at least 3 days under light-shielded conditions.

12. Remove the glass slides from PBS, aspirate the remaining PBS, and place the glass slides in a humidified chamber.
13. Wash the tissue sections twice with 50 μL of PBS.

### (Optional) Immunostaining

**Timing: 3.5–24 h**

Immunostaining is useful to visualize ROIs for subsequent photo-irradiation.

**CRITICAL:** Immunostaining protocols can largely depend on those recommended by antibody suppliers, but antigen retrieval by heating and proteinase treatment should be avoided. Overnight incubation at 4°C with primary antibodies is less harmful to the yield of sequence libraries. Secondary antibodies excited by blue (≈488 nm), green (≈555 nm), and far-red (≈633 nm) light can be used for immunolocalization, but those excited by shorter wavelengths (<425 nm) are not compatible with PIC because their excitation uncages the NPOM moieties of the RT primers.

14. Aspirate PBS from the sections, add 50 μL of blocking solution, and incubate at RT for 15 min in a humidified chamber.
15. Aspirate the blocking solution, drop 50 μL of primary antibodies diluted in blocking solution onto the section, and incubate at RT for 2 h in a humidified chamber.
16. Aspirate the primary antibody solution, wash the sections three times with 50 μL of TBST.
17. Drop 50 μL of secondary antibodies diluted in blocking solution onto the section and incubate at RT for 1 h in a humidified chamber.
18. Aspirate the secondary antibody solution and wash the sections three times with 50 μL of TBST.

### (Optional) Nuclear staining

**Timing: approximately 20 min**

Nuclear Violet is useful to observe nuclei with 426–450 nm excitation light without uncaging NPOM moieties. Nuclear Violet is also excited by ∼365 nm UV, thus acting as a useful indicator of UV-irradiated cells.

19. Aspirate the TBST from the sections, add 50 μL of Nuclear Violet LCS1 (1:500) dissolved in blocking solution, and incubate at RT for 15 min in a humidified chamber.

**CRITICAL:** Nuclear staining can be performed simultaneously during incubation with the secondary antibody (step 17).

20. Aspirate the nuclear staining solution and wash the sections three times with 50 μL of TBST.

### UV irradiation

**Timing: approximately 5 min**

NPOM moieties are uncaged by UV irradiation.

21. Aspirate the TBST from the sections and mount a round coverslip (φ15 mm) with 10 μL of SlowFade Diamond.

**CRITICAL:** Ensure that no air bubbles are present in the section. The size of the coverslip can be changed according to the size of the section, with an equivalent size being the best option.

22. Irradiate 365 nm centered UV light onto the ROIs for 3–15 min with DMD.

**Figure 4:**
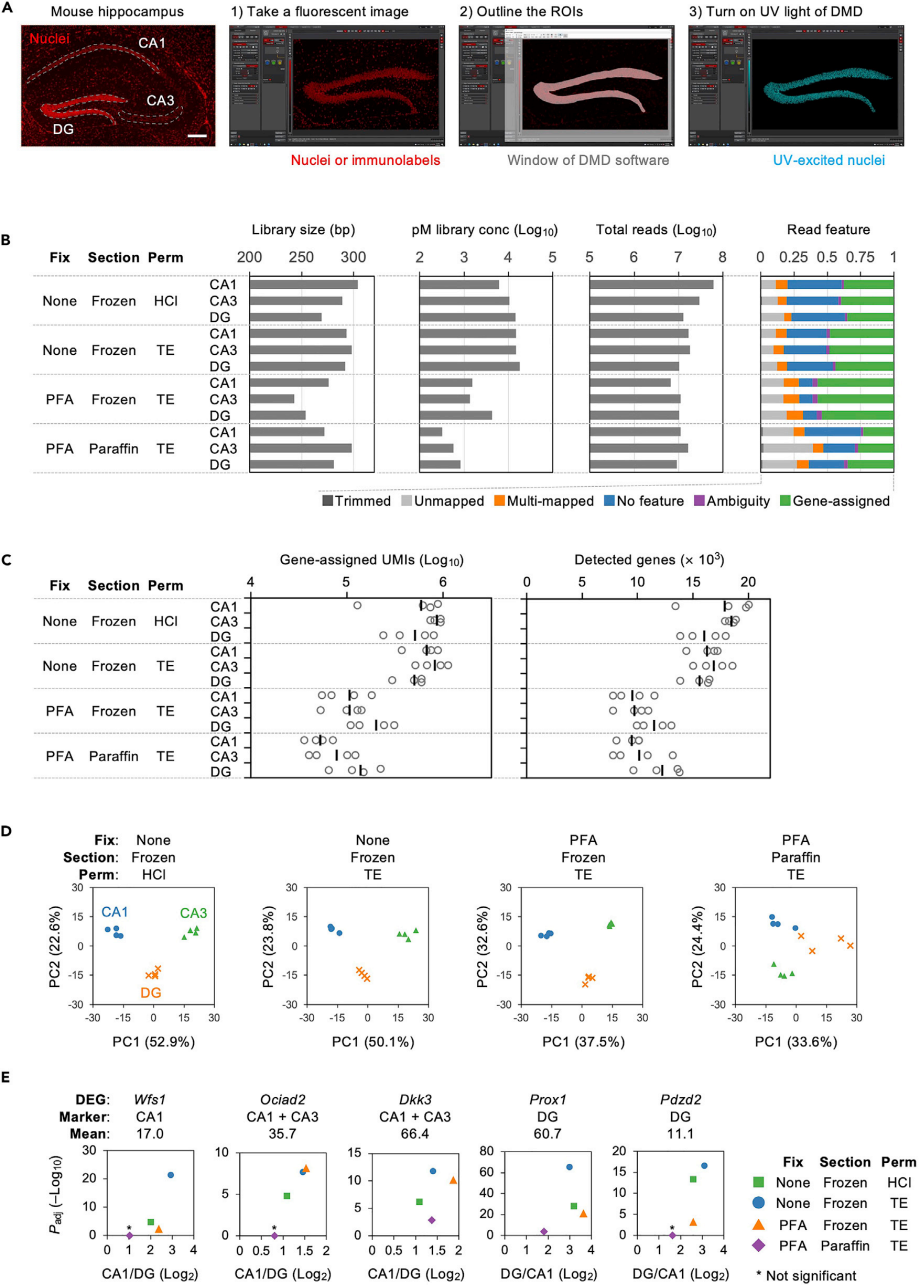
Result of PIC RNA-seq (A) Screen shots during irradiation of the DG region of mouse hippocampal tissues are shown. Scale bars, 200 μm. A nuclear staining image of DG was taken (1) and overlaid with a DMD software window to outline the ROI (2), before turning on the DMD UV light. (3). (B and C) The size and yield after the library synthesis, the number of sequenced reads, and the read features are shown in (B), with the number of gene-assigned UMIs and detected genes represented in (C) by separating the barcodes for the biological replicates. (D and E) The transcriptome data were subjected to PCA (D) and DEG analysis (E) in each histological condition.

**Figure 5:**
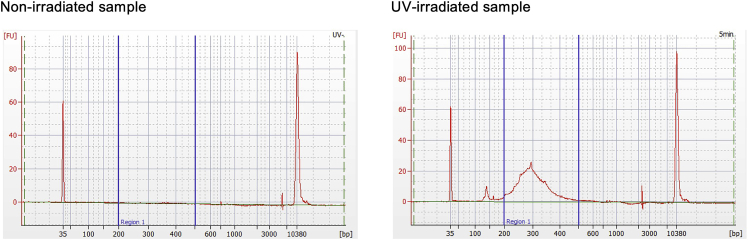
Quantification of libraries using BioAnalyzer The library quantification data with BioAnalyzer is represented for PIC analysis of mouse hippocampus with or without photo-irradiation.

**CRITICAL:** We used a Leica DM6B fluorescence microscope equipped with Polygon1000-G DMD (Mightex) and UHP-F-365 LED (Prizmatix) for the photo-irradiation (Figure 4A). First, take a fluorescent image of the nuclear staining, with immunofluorescence as optional, using Leica Application Suite X (LAS-X) software, where the excitation wavelength must be longer than 425 nm to avoid uncaging of NPOM moieties. Next, overlay a PolyScan window, software to regulate the DMD, and outline the ROIs. Then, turn on the LED light to irradiate the ROI and obtain an image of the excited nuclei to record the illuminated area. When conducting the PIC experiment for the first time, it is recommended to also prepare a non-irradiated sample and to confirm that almost no product is obtained in the subsequent library synthesis (Figure 5), which is useful to check whether unexpected uncaging reactions are prevented.

### Cell lysis

**Timing: approximately 40 min**

Whole cell lysates are collected in the test tubes.

23. Drop 300 μL of PBS onto the coverslip and remove coverslips using tweezers (Figure 3E).

**CRITICAL:** Be careful not to damage or peel tissue samples. The coverslips will float by dropping PBS onto the glass slides.

24. Wash the tissue sections twice with 50 μL of PBS.
25. Drop 40 μL of proteinase K lysis solution onto the sections and incubate for 15 min at 55°C in a humidified chamber in a hybridization oven.
26. Scrape the tissues and collect the cell lysates and debris in a 1.5 mL tube.

***Note:*** Differentially barcoded samples can be pooled in the same tube.

27. Drop 40 μL of proteinase K lysis solution onto the sections and incubate for 15 min at 55°C in a humidified chamber in a hybridization oven.
28. Collect the cell lysates and debris in the same tube as step 26 (total volume ≃80 μL).
29. Vortex and spin down (1,400 × *g*, RT, 2 s).
30. Incubate for 15 min at 55°C in a hybridization oven.
31. Vortex and spin down (1,400 × *g*, RT, 2 s).
32. Incubate for 15 min at 55°C in a hybridization oven.
33. Vortex and spin down (1,400 × *g*, RT, 2 s).

**Pause point:** The samples can be stored at −20°C for at least 1 week under light-shielded conditions.

### Purification of cDNA:mRNA hybrids

**Timing: approximately10 min**

cDNA:mRNA hybrids are purified by spin column.

34. Add 5 volumes of buffer PB from Qiagen MinElute PCR Purification kit (≈400 μL) to 1 volume of the sample (≈80 μL).
35. Vortex and spin down (1,400 × *g*, RT, 2 s).
36. Apply the lysates to MinElute Spin Column (Max 750 μL).
37. Centrifuge at 15,300 × *g* at RT for 1 min and discard flow through.
38. Add 750 μL of buffer PE to MinElute Spin Column.
39. Centrifuge at 15,300 × *g* at RT for 1 min and discard flow through.
40. Centrifuge at 15,300 × *g* at RT for 2 min and discard flow through.
41. Transfer the MinElute Spin Column to a new 1.5 mL tube.
42. Add 17 μL of nuclease-free water to the MinElute Spin Column and incubate at RT for 1 min.
43. Centrifuge at 15,300 × *g* at RT for 2 min and discard flow through.

**Pause point:** The samples can be stored at −20°C for at least 1 month under light-shielded conditions.

### Second-strand DNA synthesis

**Timing: approximately 2.5 h**

Second-strand DNA is synthesized using the nick translation method using RNase H, DNA polymerase I, and DNA ligase mix, by which only the cDNAs derived from the photo-irradiated region are filled in to the 5′ end of the T7 promoter.

44. Prepare second-strand mix, as shown in materials and equipment.
45. Mix 5 μL of second-strand mix (step 44) with 15 μL of purified cDNA:mRNA hybrids (step 43) in a 0.2 mL 8-strip PCR tube by gentle pipetting.
46. After spinning down the sample tube (1,400 × *g*, RT, 2 s), incubate the tubes at 16°C in a thermal cycler for 2 h and immediately place the tubes on ice.
47. Mix Ampure Beads and Beads Binding Buffer in a ratio of 1:5 and vortex thoroughly.
48. Add 24 μL of Ampure Beads and Beads Binding Buffer mix (step 47) to the second-stranded DNA sample (step 46) and vortex thoroughly.

**CRITICAL:** The volume of the Ampure Beads and Beads Binding Buffer mix (step 47) to be added is 1.2 times the volume of the second-stranded DNA sample (step 46). Different barcoded samples can be pooled in the same tube in this step.

49. After spinning down the sample tube (1,400 × *g*, RT, 2 s), incubate the tubes at RT for 15 min.
50. Place the sample tube on a magnetic stand and wait for at least 5 min.

**CRITICAL:** The waiting time depends on the performance of the magnetic stand. Make sure the beads are fully bonded to the magnet of the stand.

51. Remove the supernatant.

**CRITICAL:** Be careful not to aspirate the beads.

52. Add 200 μL of 80% ethanol and incubate at RT for 30 s.
53. Remove the supernatant.
54. Add 200 μL of 80% ethanol and incubate at RT for 30 s.
55. Remove the supernatant and spin down the sample tube (1,400 × *g*, RT, 2 s).
56. Place the sample tube on the magnetic stand and incubate at RT for 1 min.
57. Remove the supernatant and air dry for 2 min.

**CRITICAL:** Ensure that no ethanol remains in the tube.

58. Suspend the beads with 6.4 μL of nuclease-free water by pipetting and incubate at RT for at least 2 min in the non-magnetic stand.

### IVT

**Timing: 15–17 h (overnight reaction)**

cDNAs derived from photo-irradiated regions are linearly amplified as RNAs (aRNAs) via IVT reaction.

59. Prepare IVT mix, as shown in materials and equipment.
60. Add 9.6 μL of IVT mix (step 59) to the bead suspension containing second-stranded cDNA (step 58) in a 0.2 mL 8-strip PCR tube by gentle pipetting.
61. After spinning down the sample tube (1,400 × *g*, RT, 2 s), incubate the tubes at 37°C in a thermal cycler for 15–17 h and immediately place the tubes on ice.

### Purification of aRNA

**Timing: approximately 1 h**

62. Add 1 μL of TURBO DNase from the MEGAscript T7 Transcription Kit.
63. After tapping and spinning down the sample tube (1,400 × *g*, RT, 2 s), incubate the tubes at 37°C in a thermal cycler for 15 min and immediately place the tubes on ice.
64. Add 3 μL of ExoSAP-IT Express PCR Product Cleanup.
65. After tapping and spinning down the sample tube (1,400 × *g*, RT, 2 s), incubate the tubes at 37°C in a thermal cycler for 5 min and immediately place the tubes on ice.
66. Add 5.5 μL of Fragmentation Buffer and vortex thoroughly.
67. After spinning down the sample tube (1,400 × *g*, RT, 2 s), incubate the tubes at 94°C in a preheated thermal cycler for 3 min and immediately place the tubes on ice.
68. Add 2.75 μL of 0.5 mol/L EDTA Solution (pH 8.0) and vortex thoroughly.
69. After spinning down the sample tube (1,400 × *g*, RT, 2 s), place the sample tube on the magnetic stand and incubate at RT for 5 min.
70. Collect 28 μL of supernatant in a new 0.2 mL 8-strip PCR tube.
71. Add 50.4 μL of RNAClean XP beads and vortex thoroughly.

**CRITICAL:** The volume of RNAClean XP beads to be added is 1.8 times the volume of the sample.

72. After spinning down the sample tube (1,400 × *g*, RT, 2 s), incubate at RT for 10 min.
73. Place the sample tube on a magnetic stand and wait for at least 5 min.

**CRITICAL:** The waiting time depends on the performance of the magnetic stand. Make sure the beads are fully bonded to the magnet of the stand.

74. Remove the supernatant.

**CRITICAL:** Be careful not to aspirate the beads.

75. Add 200 μL of 70% ethanol and incubate at RT for 30 s.
76. Remove the supernatant.
77. Add 200 μL of 70% ethanol and incubate at RT for 30 s.
78. Remove the supernatant and spin down the sample tube (1,400 × *g*, RT, 2 s).
79. Place the sample tube on the magnetic stand and incubate at RT for 1 min.
80. Remove the supernatant and air dry for 2 min.

**CRITICAL:** Ensure that no ethanol remains in the tube.

81. Resuspend the beads with 6.5 μL of nuclease-free water by pipetting and incubate at RT for at least 2 min.
82. After spinning down the sample tube (1,400 × *g*, RT, 2 s), place the sample tube on the magnetic stand and incubate at RT for 5 min.
83. Collect 4.5 μL of supernatant in a new 0.2 mL 8-strip PCR tube.

**Pause point:** The samples can be stored at −80°C for at least 1 month after IVT.

### (Optional) Confirmation of aRNA by gel electrophoresis

**Timing: approximately 1 h**

84. The size and amount of aRNA can be confirmed using gel electrophoresis.

### Reverse transcription of aRNAs

**Timing: approximately 1.5 h**

aRNAs are further reverse-transcribed by random primers with the Illumina Read2 sequence.

85. Prepare aRNA–N6 primer mix, as shown in materials and equipment.
86. Spin down the sample tube (1,400 × *g*, RT, 2 s).
87. Incubate the tubes at 65°C in a preheated thermal cycler for 5 min to relax the secondary structure of the aRNAs and immediately place the tubes on ice.
88. Prepare the RT mix, as shown in materials and equipment.
89. Add 4 μL of RT mix (step 88) to the aRNA–N6 primer mix (step 87) and mix by gentle pipetting. After spinning down the sample tube (1,400 × *g*, RT, 2 s), incubate at 25°C for 10 min, and then at 42°C for 1 h in a thermal cycler.

**Pause point:** The samples can be stored at 4°C for at least 1 day.

### PCR amplification

**Timing: approxmately 1 h**

Sequence libraries are amplified by 11 cycles of PCR.

**CRITICAL:** The PCR cycles can be increased by 15 if the library yield is expected to be low due to the small number of target cells. More cycles of PCR will result in UMI saturation by over-amplification.

90. Prepare the library PCR mix, as shown in materials and equipment.
91. After spinning the sample tube (1,400 × *g*, RT, 2 s), amplify the samples using PCR, as shown below.

**Table:** 

Steps	Temperature	Time	Cycles
Initial Denaturation	98°C	30 s	1
Denaturation	98°C	10 s	11 cycles
Annealing	60°C	30 s	
Extension	72°C	30 s	
Final extension	72°C	10 min	1
Hold	4°C	forever

**Pause point:** The samples can be stored at −20°C for at least 1 month.

92. Add 1 μL of RNase A (10 mg/mL).
93. After tapping and spinning down the sample tube (1,400 × *g*, RT, 2 s), incubate the tubes at 37°C in a thermal cycler for 30 min.

### Size selection of sequence libraries (1st round)

**Timing: approximately 1 h**

**CRITICAL:** At least two rounds of size selection are recommended. Perform more rounds if the sizes do not fall within 200–400 bp.

94. Add 6 μL of nuclease-free water to the PCR products and adjust to 50 μL.
95. Mix 50 μL of PCR products with 32.5 μL of Ampure XP beads and vortex thoroughly.
96. After spinning down the sample tube (1,400 × *g*, RT, 2 s), incubate at RT for 15 min.
97. Place the sample tube on a magnetic stand and wait for at least 5 min.

**CRITICAL:** The waiting time depends on the performance of the magnetic stand. Make sure the beads are fully bonded to the magnet of the stand.

98. Collect the supernatant in a new 0.2 mL 8-strip PCR tube.
99. Add 12.5 μL Ampure XP beads, and vortex thoroughly.
100. After spinning down the sample tube (1,400 × *g*, RT, 2 s), incubate at RT for 10 min.
101. Place the sample tube on a magnetic stand and wait for at least 5 min.

**CRITICAL:** The waiting time depends on the performance of the magnetic stand. Make sure the beads are fully bonded to the magnet of the stand.

102. Remove the supernatant.

**CRITICAL:** Be careful not to aspirate the beads.

103. Add 200 μL of 80% ethanol and incubate at RT for 30 s.
104. Remove the supernatant.
105. Add 200 μL of 80% ethanol and incubate at RT for 30 s.
106. Remove the supernatant and spin down the sample tube (1,400 × *g*, RT, 2 s).
107. Place the sample tube on the magnetic stand and incubate at RT for 1 min.
108. Remove the supernatant and air dry for 2 min.

**CRITICAL:** Ensure that no ethanol remains in the tube.

109. Suspend the beads with 32 μL of nuclease-free water by pipetting and incubate at RT for at least 2 min.
110. After spinning down the sample tube (1,400 × *g*, RT, 2 s), place the sample tube on the magnetic stand and incubate at RT for 5 min.
111. Collect 30 μL of supernatant in a new 0.2 mL 8-strip PCR tube.

**Pause point:** The samples can be stored at −20°C for at least 6 months.

### Size selection of sequence libraries (2nd round)

**Timing: approximately 1 h**

112. Mix 30 μL of the sample (step 117) with 19.5 μL of Ampure XP beads and thoroughly vortex.
113. After spinning down the sample tube (1,400 × *g*, RT, 2 s), incubate at RT for 15 min.
114. Place the sample tube on a magnetic stand and wait for at least 5 min.

**CRITICAL:** The waiting time depends on the performance of the magnetic stand. Make sure the beads are fully bonded to the magnet of the stand.

115. Collect the supernatant in a new 0.2 mL 8-strip PCR tube.
116. Add 7.5 μL Ampure XP beads and vortex thoroughly.
117. After spinning down the sample tube (1,400 × *g*, RT, 2 s), incubate at RT for 10 min.
118. Place the sample tube on a magnetic stand and wait for at least 5 min.

**CRITICAL:** The waiting time depends on the performance of the magnetic stand. Make sure the beads are fully bonded to the magnet of the stand.

119. Remove the supernatant.

**CRITICAL:** Be careful not to aspirate the beads.

120. Add 200 μL of 80% ethanol and incubate at RT for 30 s.
121. Remove the supernatant.
122. Add 200 μL of 80% ethanol and incubate at RT for 30 s.
123. Remove the supernatant and spin down the sample tube (1,400 × *g*, RT, 2 s).
124. Place the sample tube on the magnetic stand and incubate at RT for 1 min.
125. Remove the supernatant and air dry for 2 min.

**CRITICAL:** Ensure that no ethanol remains in the tube.

126. Suspend the beads with 15 μL of nuclease-free water by pipetting and incubate at RT for at least 2 min.
127. After spinning down the sample tube (1,400 × *g*, RT, 2 s), place the sample tube on the magnetic stand and incubate at RT for 5 min.
128. Collect 12 μL of supernatant in a new 0.2 mL 8-strip PCR tube.

**Pause point:** The samples can be stored at −20°C for at least 6 months.

### Quantification of the library

**Timing: 1–2 h**

129. Measure the size distribution of the PIC library using a bioanalyzer by loading 1 μL on a chip in a High Sensitivity DNA Kit, according to the manufacturer’s instructions.

**CRITICAL:** The expected size is 200–400 bp (Figure 5), otherwise repeat the size selection (steps 100–134).

### Sequencing and data analysis

**Timing: ≥1 day**

130. Perform paired-end sequencing on an Illumina sequencer (for example, HiSeq or NovaSeq) following the manufacturer’s instructions. We usually add PhiX at 5% for the final spike in and take 10–20 million reads (Read1, 12 cycles for UMI and barcode 18; Read2, ≥50 cycles for cDNA), which may vary depending on the ROI area and library yield.
131. Extract the barcodes and UMIs in the reads using UMI-tools with the following command:

umi_tools extract -I read1.fastq --read2-in=read2.fastq --bc-pattern=NNNNNNCCCCCC --read2-stdout

132. Trim the adapter sequence from the reads using Trim Galore! (version 0.6.6) with the following command:

trim_galore -a GATCGTCGGACT

133. Map the reads by aligning the HISAT2 software to the appropriate reference genome (GRCm38, in this case).
134. Determine the read counts per gene with featureCounts and UMI-tools with the following command:

featureCounts -a GRCm38.gtf -R BAM input.bam

samtools index input.featureCounts.bam

umi_tools count --method=unique --per-gene --per-cell --gene-tag=XT -I input.featureCounts.bam

135. Using the resulting counts, extract differentially expressed genes (DEGs) using the R library DESeq2, which is also used to transform the count data into regularized log data before performing principal component analysis (PCA) using the R prcomp function.

## Expected outcomes

In the present experiment, we prepared fresh-frozen sections and formalin-fixed frozen and paraffin sections from the adult mouse hippocampus. HCl or TE was adopted in the permeabilization step for the fresh-frozen sections, whereas TE alone was used for the other sections. After performing *in situ* RT with NPOM-caged RT primer, CA1, CA3, or DG regions of the hippocampus were photo-irradiated with DMD (Figure 4A; n = 4 biological replicates for each condition). After the library synthesis, BioAnalyzer analysis showed that the size of the library was centered to 250 bp, and fresh-frozen sections yielded an average of 10.4 and 15.8 nM for HCl or TE treatment, respectively, in the final volume of 12 μL (Figure 4B). However, the yields of formalin-fixed tissues were lower, 2.4 nM for frozen sections and 0.57 nM for paraffin sections. Sequencing results showed that all samples provided approximately 1 × 10^7^ reads, and about half of them in the frozen sections were assigned to the genes, as indicated in a benchmark paper for CEL-seq2 (Mereu et al., 2020), while slightly fewer were assigned for the paraffin sections (Figure 4B). The number of gene-assigned UMIs and the genes detected were better in unfixed tissues (averaging 6.5 × 10^5^ UMIs / 17,392 genes and 6.5 × 10^5^ UMIs / 16,186 genes in HCl or TE-permeabilized tissues, respectively) relative to formalin-fixed frozen or paraffin sections (1.4 × 10^5^ UMIs / 10,190 genes or 8.9 × 10^4^ UMIs / 10,548 genes, respectively; Figure 4C). The UMI count data were normalized with DESeq2 and subjected to PCA using prcomp in the R package, in which all samples were separated into three groups according to the photo-irradiated regions (CA1, CA3, and DG; Figure 4D). DEG analysis was conducted with DESeq2 by comparing CA1 and DG in each condition and showed that known marker genes (Cembrowski et al., 2016) were detected as DEGs in the frozen sections despite fixation (Figure 4E; *Wfs1* [CA1 marker], *Ociad2* and *Dkk3* [CA1 and CA3 markers], *Prox1*, and *Pdzd2* [DG markers]). In contrast, in paraffin sections, genes with high average expression (*Dkk3* and *Prox1*) were detected as statistically significant DEGs, whereas genes with low average expression (*Wfs1*, *Ociad2*, and *Pdzd2*) were not, but the fold changes were all oriented toward the expected direction (Figure 4E). These results indicate PIC can isolate transcriptome information from the photo-irradiated region not only in fresh-frozen sections but also in formalin-fixed frozen or paraffin sections. Because the formalin-fixed samples were inferior to fresh-frozen samples in terms of the number of detected genes and the detection sensitivity of DEGs, it is expected these will provide better performance by sequencing a larger number of reads and by preparing more biological replicates for the experiment.

## Limitations

In the PIC method, multiple ROIs can be irradiated simultaneously, but they cannot be separated as different samples. A total of 3 × 10^6^ reads is often sufficient for sequencing analysis, but 1–2 × 10^7^ reads are recommended for small ROIs and/or for comparison between ROIs with similar characteristics. Empirically, a transcriptome of sufficient depth can be obtained with over 100 cells, but we have experienced that library yields are much lower in some tissues, such as the myocardium and aorta. Therefore, it is necessary to consider the number of cells to be irradiated for the tissues being analyzed for the first time. The first step was to irradiate 100 cells and monitor the library synthesis using a BioAnalyzer. If the library yield is lower than desired, the number of irradiated cells should be increased.

## Troubleshooting

### Problem 1

No/reduced immunostaining (step 22).

### Potential solution

Loss of antigens: Increase the fixation time to prevent dissipative loss of antigens (see Figure 1).

Poor antibody specificity: Test with an antibody that does not require antigen retrievals or is reactive to the antigens even after permeabilization with HCl or TE-heating.

### Problem 2

No/reduced library yield.

### Potential solution

Degraded RNA: Frozen tissues should be stored at −80°C, but store paraffin blocks at 4°C or lower. After sectioning, proceed with PIC experiment immediately or store the sections at −80°C.

Over-fixation: Reduce the fixation time or use fresh-frozen tissues to decrease the cross-linkage of mRNAs. Empirically, even fixation for 10 min improves preservation of tissue morphology of small organs and embryos, and fixation for 6 h effectively prevents dissipative antigen loss (Figure 1B).

Low cell number for ROIs: Try to irradiate 100 or more cells (step 22). In addition, barcoded *in situ* RT (step 3) and pooling (step 26) of multiple samples can reduce the loss in subsequent library synthesis.

Insufficient uncaging: Maximize the power of the light source and increase photo-irradiation time (step 22; Figure 2F).

### Problem 3

Background signal from non-irradiated samples.

### Potential solution

Unexpected uncaging: Order an OPC-grade purification rather than an HPLC purification (see “synthesis of caged RT primer” in before you begin). Ensure that the samples are shielded from light when storing the caged primer and conducting the extended period incubation steps, such as *in situ* RT, antibody reactions, cell lysis, and second-strand DNA synthesis (steps 8, 14, 15, 17, 19, 25, 27, 30, and 32).

### Problem 4

Decreased detection of DEGs (step 135).

### Potential solution

Low number of replicas: For each sample group, prepare at least 3–4 replicates and more, if possible, to increase statistical power.

Contamination by unexpected uncaging: Ensure that the samples are light shielded, as noted above. When observing the nuclear staining and immunostaining images, make sure to excite with ≥425 nm wavelength and ensure that the DMD light source is turned off.

## Resource availability

### Lead contact

Further information and requests for resources and reagents should be directed to and will be fulfilled by the lead contact, Shinya Oki (oki.shinya.3w@kyoto-u.ac.jp).

### Materials availability

All materials are available commercially.

## Data Availability

The datasets generated during our study are available on GEO with accession number GSE195459 and are referenced in the original study.
